# Questioning the long-term stability of the additive model in comorbid CTD+ADHD - The transition from childhood to adulthood

**DOI:** 10.1371/journal.pone.0207522

**Published:** 2018-11-20

**Authors:** Olga Müller, Aribert Rothenberger, Geza L. Brüni, Biyao Wang, Andreas Becker

**Affiliations:** 1 Clinic for Child and Adolescent Psychiatry and Psychotherapy, University Medical Center, Göttingen, Germany; 2 Service for Child and Adolescent Psychiatry Thurgau, Romanshorn, Switzerland; University of Bern, SWITZERLAND

## Abstract

**Background:**

A previous study (Roessner et al. 2007) found psychopathological evidence of an additive model of the comorbid group with Chronic Tic Disorders and Attention Deficit Hyperactivity Disorder (CTD+ADHD), which demanded clinical interventions aimed primarily at the factor ADHD. This 14-year follow-up study tested whether this childhood additive model can also be found in young adulthood and whether ADHD remains the most impairing factor.

**Methods:**

92 patients (22.8% girls) from Roessner et al. (2007) were re-investigated as young adults at the age of 24 years, broken down into four groups: CTD-only (n = 22), CTD+ADHD (n = 23), ADHD-only (n = 24), and controls (n = 23). The Adult Behavior Checklist (ABCL) was used as an equivalent parent-report instrument to the Child Behavior Checklist (CBCL) applied 14 years ago. Statistically, 2x2 factorial design was completed.

**Results:**

From the point of view of parents, the factors CTD and ADHD in young adults contributed almost equally to psychopathological problems and showed many interactions, i.e. an interactive model was supported. In addition, the ADHD factor was no longer the leading problem for psychosocial impairment in the adult CTD+ADHD group.

**Conclusion:**

The additive model of CTD+ADHD seems to exist no longer in young adults, nor may the childhood predominance of the factor ADHD in comorbid CTD+ADHD. Thus, treatment priority should be decided by clinicians on a case-by-case basis depending on the most impairing disorder of each patient.

## Introduction

Chronic Tic Disorders (CTD) and Attention Deficit Hyperactivity Disorder (ADHD) are disabling neuropsychiatric disorders with childhood onset and development into adulthood, where their symptom profile and prevalence rates may have changed. Both disorders show a high rate of comorbidity; about 80% of individuals present with at least one additional behavioral problem [1, 2].

While ADHD co-exists in about 50 percent of patients with CTD, around 20% of patients with ADHD also have CTD. Both of these percentages are well above the level of chance [3].

The phenomenological relationship between CTD and ADHD, especially in the long-term, appears complex. While in CTD most cases show a remitting course [4], the negative long-term effects of ADHD, such as clinically significant psychopathology and psychosocial dysfunction in later life, have to be taken into account in many patients. Clinical research has shown that the predominant features of ADHD in adults differ from those of ADHD in children: the symptom cluster of hyperactivity and impulsivity declines over time, while the symptoms of inattention largely persist [5, 6]. CTD+ADHD in childhood generally shows higher levels of psychopathology compared with levels in children with a single of the two disorders. In CTD+ADHD in children and adolescents, the ADHD factor is strongly related to externalizing as well as internalizing symptoms, whereas CTD is related to internalizing psychopathology only, specifically to somatic complaints [7]. Using a 2x2 factorial design, the psychopathological study by Roessner et al. [7] strongly supports the existence of an additive model of the factors CTD and ADHD. This implies that tics and comorbid ADHD are two separate diagnostic entities [8]. Therefore, it is suggested that both ADHD and CTD should be treated in parallel. However, Roessner et al.’s [7] results also revealed ADHD to have greater importance than CTD in the psychopathology of the comorbid group, prompting them to suggest that the successful treatment of ADHD should be the main focus of therapy for patients with comorbid CTD and ADHD. Although it has been shown that some childhood impairments caused by ADHD still persist into adulthood [9, 10], the question remains as to whether the clinical meaning of ADHD as part of the co-occurrence of CTD and ADHD might be different after the transition from childhood to adulthood, especially since both symptomatologies appear to decline with age [11]. This requires clarification, in terms of whether the additive model for CTD+ADHD psychopathology in childhood can also be found in adulthood, and whether for young adults ADHD remains the earlier recommended primary factor for clinical intervention. Therefore, in the present study we conducted a 14-year follow-up of Roessner et al.’s [7] study in order to examine whether the additive model for the comorbid psychopathology of CTD+ADHD found in childhood could also be detected in young adulthood, and whether ADHD is still the most impairing factor.

## Methods

### Participants

As described in more detail in Roessner et al. [7], 467 participants (24.5% girls, more than 99% Caucasian) were referred to the center of excellence for CTD, ADHD, and OCD at the Clinic for Child and Adolescent Psychiatry and Psychotherapy at the University Medical Center of Goettingen, Germany, between 1997 and 2005. They were divided into three patient groups: CTD-only *n* = 112, mean age = 11.1, *SD* = 2.6 years; CTD+ADHD *n* = 82, mean age = 10.7, *SD* = 2.3 years; and ADHD-only *n* = 129, mean age = 10.5, *SD* = 2.5 years. A control group (*n* = 144, mean age = 10.4, *SD* = 2.4 years) was made up of children from the general outpatient clinic (43% with a learning disorder, 57% with no diagnosis). Patients with CTD and/or ADHD fulfilled the diagnostic criteria for these disorders according to the Diagnostic and Statistical Manual of Mental Disorders DSM-IV-TR [12] as well as the International Classification of Diseases ICD-10 [13]. All diagnoses were verified in a case conference by senior board-certified child and adolescent psychiatrists who have been working in clinical and research settings for CTD and ADHD for many years. The follow-up investigation was conducted in 2016. In the first step, all 467 patients in the first study were contacted by mail, explaining the follow-up study and inviting for participation. This invitation letter was accompanied by a prepared response letter and a text for written consent to be signed by mail together with written consent. 92 patients (22.8% girls) responded and all of them agreed to complete the follow-up questionnaires, we could not get a response from the rest of patients, either because change of address during the long interval of 14 year or their unwillingness of responding to the follow-up investigation. Of those who responded to the follow-up investigation, there was no refusal. In a second step, families were contacted by phone for further clarification. Since no repeat formal clinical assessment was carried out, the groups were created according to their former diagnoses at baseline with all participants retaining in the same groups as reported in Roessner et al. [7]. The mean age of the total sample was 24.1 years (*SD* = 2.6, range 17–33 years), More specifically, for the CTD-only group, *n* = 22, mean age = 23.9 years, *SD* = 1.7 years; for the CTD+ADHD group, *n* = 23, mean age = 24.4 years, *SD* = 1.8 years; for the ADHD-only group, *n* = 24, mean age = 24.4 years, *SD* = 3.2 years; and for the control group, *n* = 23, mean age = 24.1 years, *SD* = 3.1 years. Both investigations were approved by the local ethics committee (Ethik-Kommission der Universitätsmedizin Göttingen) and written informed consent was given by all participants (baseline investigation from parents, follow-up investigation from both parents and patients).

### Test of bias

Given that our retention rate was only about 20% (*n* = 92) of the original group (*N* = 467), we first tested whether this follow-up-group was representative of the patients in the previous study. Information concerning age, gender, and psychopathological level at the time of the original study was collected for the follow-up group and compared with that of the original group as a whole. We also tested whether the additive model for childhood CTD+ADHD was apparent in the follow-up group at the time of the first investigation. That the follow-up group (*n* = 92) was representative of the original group and showed an additive model for childhood CTD+ADHD were considered prerequisites for this investigation.

### Psychopathological measures

In order to gather data for a dimensional broad-band psychopathological profile, the well-known and psychometrically sound ASEBA (Achenbach System of Empirically Based Assessment) instruments were used [14, 15]. In the original study, the Child Behavior Checklist (CBCL) had been completed by the parents because the children were too young to self-report. In our young adulthood study, the Adult Behavior Checklist (ABCL) was completed by parents. The inventory generates scores on eight subscales, namely: anxious/depressed, withdrawn, somatic complaints, thought problems, attention problems, aggressive behavior, rule breaking behavior, and social problems/intrusive problems. The subscale of social problems in the CBCL was replaced by the subscale of intrusive problems in the ABCL. All items were rated on a three-point scale, and then T-scores of each scale generated. The CBCL and ABCL respectively represent the childhood (4–16 years) and adulthood (18–59 years) version of the same inventory, and thus can validly be used for examining the development of psychopathology over time [16, 17].

### Statistics

In the a 2x2 factorial design study by Roessner et al. [7], two dummy variables were created to reflect the factors CTD and ADHD (no = 0, yes = 1) in patients with CTD and/or ADHD according to their clinical diagnosis at baseline, i.e., CTD-only (1, 0), CTD+ADHD (1,1), ADHD-only (0,1), controls (0,0). In this study, univariate analyses of variance (ANOVA) were conducted to test whether the additive model of CTD+ADHD in childhood could also be found in young adulthood, using the parent reports (ABCL). Full models were tested using the ABCL subscale scores as dependent variables, and the factors CTD, ADHD, and their interaction as independent variables. A lack of a significant interaction between CTD and ADHD would support the existence of an additive model in which the comorbid CTD + ADHD in children represent a combination of two independent pathologies. Such model suggested that the psychopathology of patients with co-existing ADHD and CTD diagnosis is impacted by symptoms of CTD in addition to symptoms of ADHD in a sumative linear manner (for further explanation of additive effects see Burstein et al.[18]; Rothenberger et al.[19]). It is worth pointing out that the existence of an interactive model would, on the contrary, suggest the comorbid CTD + ADHD in children represent a separate nosological entity manifested by both tics and ADHD symptoms. If the existence of an additive model was supported, then the relative contribution of factor CTD and factor ADHD to the psychopathology in the comorbid group would be computed and compared. The calculation was performed on the assumption that the difference in the sum of mean values from the CBCL subscales between children with and without a tic disorder does not deviate from the difference in the sum of mean values in children with and without ADHD ((ADHD / CTD + CTD)—(ADHD + controls)) = (ADHD / CTD + ADHD)—(CTD+ controls)). After solving the equations the following contrasts were weighted when entered into the model: controls = 0; CTD = 1; ADHD = -1 and CTD/ADHD = 0.

All the statistical analyses described above were conducted using SPSS (version 24.0).

### Problems of bias

Detailed characteristics at the time of the original study (baseline) were described and compared among the follow-up group of *n* = 92 (subsample), and the original group of *N* = 467 (total sample).

The mean age of the follow-up group at baseline was 10.2 years (*SD* = 2.3) with no significant difference compared to the entire original group (*t* = -1.512, *df* = 91, *p* = .13). For the CTD and/or ADHD groups specifically, mean age at baseline was 10.2 years (*SD* = 2.4) for CTD-only, 10.3 years, (*SD* = 1.7) for CTD+ADHD, 9.5 years (*SD* = 2.2) for ADHD-only, and 11.1 years (*SD* = 2.6) for controls. We found no significant age differences between the follow-up group and the entire original group with CTD+ADHD (*t* = -0.936, *df* = 22, *p* = .36) or the control group (*t* = 1.262, *df* = 22, *p* = .22), and only marginal age differences in the CTD-only (*t* = -2.004, *df* = 21, *p* = .06) and ADHD-only (*t* = -2.110, *df* = 23, *p* = .05) groups.

There was also no gender difference between the follow-up group and the entire original group (χ^2^ [*df* = 1] = 0.47, *p* = .50). Even after CTD and/or ADHD groupings were considered, similar results were found: that is, no significant gender differences were found for the CTD-only (χ^2^ [*df* = 1] = 0.02, *p* = .89), CTD+ADHD (χ^2^ [*df* = 1] = 0.01, *p* = .97), ADHD-only (χ^2^ [*df* = 1] = 0.30, *p* = .59), or control (χ^2^ [*df* = 1 ] = 1.23, *p* = .27) groups.

For each CTD and/or ADHD group, the mean scores of each CBCL at baseline were compared between the follow-up group and the entire original group. The CTD and control follow-up groups showed no differences from the entire original group in mean scores on any of the subscales. However, the ADHD follow-up group reported higher levels of aggression (*t* = 3.506, *df* = 23, *p* < .01), delinquency (*t* = 2.607, *df* = 23, *p* < .05), attention problems (*t* = 3.993, *df* = 23, *p* < .01), thought problems (*t* = 2.676, *df* = 23, *p* < .05), and being withdrawn (*t* = 2.405, *df* = 23, *p* < .05), while the CTD+ADHD follow-up group reported higher levels of attention problems (*t* = 4.919, *df* = 22, *p* < .01), anxiety/depression (*t* = 3.864, *df* = 22, *p* < .01), thought problems (*t* = 4.099, *df* = 22, *p* < .01), withdrawn (*t* = 3.696, *df* = 22, *p* < .01), and social problems (*t* = 3.494, *df* = 22, *p* < .01). The altered psychopathological level showed that the follow-up group of ADHD and CTD+ADHD had more severe problems at baseline than the original group. Considering ADHD was the most impairing factor in the childhood additive model in comorbid CTD+ADHD (Roessner et al. 2007) and the only shared factor of those groups, the increase of psychopathology in the follow-up group at the starting point may also stem from the factor ADHD and therefore may suggest that the follow-up group was even more impaired by the factor ADHD in childhood than the original group.

More importantly, we tested whether the additive model for childhood CTD+ADHD could also be found in the smaller (compared with the original group of *N* = 467) follow-up group of *n* = 92 at baseline. As shown in Table 1, there was no significant interaction between the factors CTD and ADHD, with one exception: the subscale anxious/depressed showed a very small effect size (*η*^*2*^ = .09). These results support the finding of an additive model at first investigation for childhood CTD+ADHD also in our follow-up group of *n* = 92, similar to that found by Roessner et al. (2007) for the entire group. The subsequent comparison of main factors in Table 2 showed that the factor ADHD was related more strongly, or at least equally, to every CBCL subscale except for the anxious/depressed subscale for which the contrast could not be performed because of significant interaction between factors. These results also replicate those of Roessner et al. [7], who drew similar conclusions on the basis of the entire original group.

**Table 1:** 2x2 ANOVA of the eight child behavior checklist subscales for the factors ADHD and CTD and descriptive measures (baseline; n = 92 from 467).

	CTD-only (1) (*n* = 22)	CTD +ADHD (2) (*n* = 23)	ADHD-only (3) (*n* = 24)	CONTROL (4) (*n* = 23)	ANOVA F (1,88)								
	M SD	M SD	M SD	M SD	CTD (η^2^)			ADHD (η^2^)			ADHD x CTD (η^2^)		
**Aggressive Behavior**	57.6 (7.3)	67.5 (10.3)	71.1 (6.4)	54.8 (5.6)	0.08		.01	67.87	***	.44	2.07		.01
**Delinquent Behavior**	55.5 (7.3)	64.4 (9.8)	68.6 (8.1)	55.0 (6.7)	1.29		.01	44.63	***	.34	1.90		.02
**Attention Problems**	62.0 (5.7)	76.4 (5.2)	73.5 (8.1)	60.1 (4.2)	5.1	*	.03	170.86	***	.66	0.21		.01
**Anxious/ Depressed**	60.0 (6.4)	70.8 (6.6)	59.9 (7.0)	57.2 (6.7)	24.45	***	.22	22.94	***	.21	8.31	*	.09
**Thought Problems**	58.9 (7.1)	67.8 (6.7)	62.2 (8.5)	53.6 (5.1)	15.23	***	.15	37.34	***	.30	0.01		.01
**Withdrawn**	58.8 (7.5)	63.6 (4.8)	63.2 (7.6)	57.7 (7.9)	0.02		.01	14.33	***	.14	0.01		.00
**Somatic Complaints**	56.9 (7.1)	61.4 (8.2)	57.6 (7.3)	54.3 (5.5)	4.57	*	.05	6.71	*	.07	0.14		.01
**Social Problems**	58.9 (7.9)	72.7 (9.3)	66.1 (11.4)	55.7 (7.1)	6.83	*	.08	40.89	***	.32	0.37		.01

*Note*: M = mean, SD = standard deviation. Effects are from 2 x2 ANOVA with ADHD and CTD as factors. The effect sizes presented in Table 1 are the partial eta squared produced by the ANOVA. Cohen (1977) provides the following guidelines for interpreting the eta squared *(η*^*2*^) value: .01 - .059 = small effect size; .06 - .139 = medium effect size; > .14 = large effect size.

***p < .001

**p < .01

*p < .05

**Table 2:** Comparison of the main factors (baseline; n = 92 from 467).

CBCL subscale	contrast score	t (88)		factor comparison^a^
**Aggressive Behavior**	-27.07	-6.61	***	ADHD > CTD
**Delinquent Behavior**	-26.34	-5.84	***	ADHD > CTD
**Attention Problems**	-23.00	-6.55	***	ADHD > CTD
**Anxious/ Depressed** ^**b**^	—	1.33		—
**Thought Problems**	-6.42	-1.56		ADHD = CTD
**Withdrawn**	-10.87	-2.41	*	ADHD > CTD
**Somatic Complaints**	- 1.34	-0.31		ADHD = CTD
**Social Problems**	-14.35	-2.49	*	ADHD > CTD

*Note*: CBCL = Child Behavior Checklist (Achenbach, 1991).

***p < .001

*p < .05

^a^ The calculation was performed on the assumption that the difference in the sum of mean values from the CBCL subscales between children with or without a tic disorder does not deviate from the difference in the sum of mean values in children with or without ADHD ((ADHD/CTD + CTD)—(ADHD + Control) = (ADHD/CTD + ADHD)—(CTD + control)). After solving the equations the following contrast weights resulted: Control group = 0; CTD = 1; ADHD = -1 and CTD/ADHD = 0.

^b^ Contrasts could not be computed due to the significant interaction in the ANOVA.

The above results show that despite the bias of somewhat higher general psychopathology by the factor ADHD, the follow-up group of *n* = 92 is still representative enough of the original group of *N* = 467 to clarify the relationship between CTD and ADHD. This representativeness allowed us to test for a corresponding additive model of CTD+ADHD in young adulthood using the comparable ABCL from parent report.

## Results

### Descriptive information

In the follow-up group of n = 92, there were no significant age [F(3,88) = 1.83, p = 0.15] or gender [F(3,88) = 2.10, p = 0.11] differences between the CTD and/or ADHD groups.

From a descriptive point of view, most mean scores on the ABCL subscales (in Table 3) were lower than those on the CBCL (Table 1), indicating a general improvement in symptoms from childhood to adulthood. However, in keeping with the findings from childhood, the young adulthood comorbid group with CTD+ADHD still reported the highest scores of the four CTD and/or ADHD groups, followed by the ADHD-only group, while the CTD-only and control groups reported more or less similar scores; i.e. the groups improved more or less in parallel while their group ranking remained the same.

**Table 3:** 2x2 ANOVA of the eight ABCL-syndrome scales for the factors CTD and ADHD and descriptive measures (follow-up; n = 92).

	*CTD-only* *(n = 22)*	*CTD +ADHD* *(n = 23)*	*ADHD-only* *(n = 24)*	*CONTROL* *(n = 23)*	*ANOVA* *F (1*,*88)*								
	M SD	M SD	M SD	M SD	CTD (η^2^)			ADHD (η^2^)			ADHD x CTD (η^2^)		
**Aggressive Behavior**	52.3 (3.5)	63.4 (8.4)	55.4 (5.4)	53.4 (4.7)	8.11	**	.10	24.85	**	.24	13.84	***	.15
**Rule Break. Behavior**	52.9 (3.3)	65.1 (12.6)	56.2 (6.2)	53.8 (4.4)	6.09	*	.07	20.17	***	.21	9.21	**	.11
**Attention Problems**	54.1 (5.1)	66.9 (10.2)	59.0 (6.9)	53.9 (4.6)	6.75	*	.08	33.58	***	.30	6.41	*	.08
**Anxious/ Depressed**	52.5 (4.5)	60.7 (11.2)	55.0 (5.1)	54.5 (5.9)	1.41		.02	7.90	**	.09	6.13	*	.07
**Thought Problems**	52.4 (3.6)	58.8 (10.0)	54.0 (6.2)	51.0 (2.3)	5.26	*	.06	11.93	***	.13	1.56		.02
**Withdrawn**	54.4 (5.9)	63.0 (10.7)	56.0 (8.7)	54.1 (5.5)	4.10	*	.05	8.81	**	.10	3.60		.05
**Somatic Complaints**	54.0 (5.4)	59.0 (7.6)	52.0 (3.8)	52.4 (3.3)	14.03	***	.15	4.02	*	.05	5.84	*	.07
**Intrusive**	53.0 (3.6)	56.6 (5.3)	53.7 (5.5)	52.0 (3.4)	3.63		.05	6.80	*	.08	0.88		.01

*Note*: *M = mean*, *SD = standard deviation*. *Effects are from 2 x2 ANOVA with CTD and ADHD as factors*. *The effect sizes presented in Table 3 are the partial eta squared* produced by the ANOVA. Cohen (1977) provides the following guidelines for interpreting the eta squared *(η*^*2*^) value: .01 - .059 = small effect size; .06 - .139 = medium effect size; > .14 = large effect size.

***p < .001

**p < .01

*p < .05

### Testing the additive model

The additive model for CTD+ADHD in adulthood was first tested using information from the parents (ABCL scores). In the 2x2 ANOVA, scores on the eight ABCL syndrome subscales were used as dependent variables, the factors CTD and ADHD as independent variables. The full model resulted in five significant interaction effects between the factors CTD and ADHD on the subscales of aggressive behavior, rule breaking behavior, attention problems, anxious/depressed, and somatic complaints (Table 3). Contrasts were calculated only for the three subscales in which no significant interaction effects between CTD and ADHD were found. On these three subscales—“thought problems,” “withdrawn,” and “intrusive problems”—the factors CTD and ADHD contributed equally to the psychopathological symptoms (Table 4). Hence, parental information supported an interactive model, rather than an additive model, for comorbid CTD+ADHD in adulthood.

**Table 4:** Comparison of the main factors ABCL (follow-up; n = 92).

ABCL-subscale	contrast score	t (88)	factor comparison^a^
**Aggressive Behavior**^**b**^	--	--	--
**Rule Break. Behavior**^**b**^	--	--	--
**Attention Problems**^**b**^	--	--	--
**Anxious/ Depressed**^**b**^	--	--	--
**Thought Problems**	-1.58	-1.04	ADHD = CTD
**Withdrawn**	-1.67	-0.75	ADHD = CTD
**Somatic Complaints**^**b**^	--	--	--
**Intrusive Problems**	-0.72	-0.52	ADHD = CTD

*Note*: ABCL = ABCL/18-59 (Adult Behaviour Checklist) (Achenbach, 2012).

***p < .001

**p < .01

*p < .05

^a^ The calculation was performed on the assumption that the difference in the sum of mean values from the CBCL subscales between children with or without a tic disorder does not deviate from the difference in the sum of mean values in children with or without ADHD ((ADHD/CTD + CTD) - (ADHD + Control) = (ADHD/CTD + ADHD) - (CTD + control)). After solving the equations the following contrast weights resulted: Control group = 0; CTD = 1; ADHD = -1 and CTD/ADHD = 0.

^b^ Contrasts could not be computed due to the significant interaction in the ANOVA.

## Discussion

The main objective of this 14-year follow-up psychopathological study was to investigate 1) whether the additive model for comorbid CTD+ADHD found in childhood could also be detected in young adulthood, and 2) whether ADHD was still the most impairing factor. From an original study of 467 patients [7], 92 patients (20%) could be re-investigated. Following a series of tests of age, gender, psychopathological characteristics, and the existence of an additive model for comorbid CTD+ADHD in childhood, the follow-up sample of *n* = 92 was found to be sufficiently representative of the original sample of *N* = 467, despite some bias, namely that the follow-up group in childhood might have been more impaired by the factor ADHD than the original group. The psychopathological profile in young adulthood of the four clinical groups—as originally assessed in childhood, namely CTD-only, ADHD-only, CTD+ADHD, and clinical controls—was assessed using parent (ABCL) ratings on the eight subscales of the ASEBA instruments. In general, the scores on these subscales decreased from childhood (CBCL) to adulthood (ABCL), i.e. after 14 years, patients presented with fewer psychopathological problems. Similar to the pattern found in childhood, the highest levels of psychopathology were found in the comorbid CTD+ADHD group, followed by the ADHD-only group. The CTD-only and control groups reported more or less similar levels. Hence, in descriptive terms, the children in the CTD and/or ADHD groups showed parallel improvement in their psychopathological symptoms in young adulthood, while retaining the same ranking they had in childhood. The higher scores on psychopathology in the comorbid group compared with those with pure CTD or ADHD correspond to previous research [3, 20, 21]. This long-term effect of psychopathological “within-group stability” is known from other longitudinal research as well, although changes for the better or the worse can be seen in a small proportion of children within their group as they grow up [22]. The existence of an additive model of CTD+ADHD in young adulthood was examined with a univariate ANOVA using ABCL subscale scores as dependent variables, and the factors CTD, ADHD and their interaction as independent variables. In contrast with the additive model of CTD+ADHD found in childhood data with CBCL rated by the same parents [7], this time. parent ratings with ABCL did not support an additive model of CTD+ADHD in young adulthood. Although there were significant main effects of CTD (six scales) and ADHD (eight scales) on most subscales, no clear separation between the factor effects could be identified for psychopathological problems as there was in childhood (i.e. CTD influenced only internalizing problems, ADHD influenced both internalizing and externalizing problems). Significant interactions between CTD and ADHD were broadly apparent on the ABCL subscales (aggressive behavior, rule breaking behavior, attention problems, anxious/depressed, and somatic complaints), supporting an interactive rather than additive model for comorbid CTD+ADHD. For the non-interactive effects where the contribution of the factors CTD and ADHD could be computed and compared, these factors were found to contribute equally to thought problems, being withdrawn, and intrusive problems. All these results suggest that, from a parental point of view, the psychopathological model of CTD+ADHD changed from an additive to an interactive one during the transition from childhood to young adulthood.

In view of these results, those parents perceived the comorbidity CTD+ADHD in young adulthood as a single disorder (interactive model), despite their observations in childhood (using the CBCL) supporting an additive model. Possible reasons for this, such as the different perspectives of the respondents or the natural course of the disorder, for example, have still to be elucidated. More longitudinal psychopathological research is needed to clarify this issue, possibly in combination with neurobiological and genetic investigations [3]. In addition, despite the follow-up group in their childhood may have been even more impaired by the factor ADHD than the original group, the childhood predominance of the factor ADHD no longer exists, i.e., the factor ADHD no longer acted as the leading clinical problem but affected patients’ psychopathological level to a similar extent as the factor CTD in young adulthood CTD+ADHD comorbidity, implying that ADHD and CTD deserve more or less equal clinical consideration in this group of patients.

### Limitations

Due to the long interval of time, 14 years, between the original investigation and this re-investigation we could only reach 20% of Roessner et al.’s [7] original sample. Through detailed analyses of age, gender, psychopathological characteristics, and the existence of an additive model of CTD+ADHD in childhood, we were able to prove that the follow-up sample was representative of the original sample. There could be possible bias in the ADHD and CTD+ADHD follow-up group, as patients in these follow-up groups reported higher scores in childhood than those of the original groups on some of the CBCL scales. Since ADHD was the most impairing factor in childhood, the follow-up ADHD and CTD+ADHD groups could be considered to be more strongly influenced by the factor ADHD than the original ADHD and CTD+ADHD groups were. This could result in a possible preference for ADHD predominance in young adulthood. However, results showed that both ADHD and CTD played a similar role concerning the psychopathological symptoms in young adulthood, even with the stronger influence of factor ADHD in childhood.

The lack of information on symptom severity and treatment is another limitation of this study, because the impact of a disorder on general psychopathology largely depends on these factors. Hence, it remains unclear to what extent the shift of an additive model to an interactive model may reflect the natural clinical course of CTD and ADHD or the effect of treatment. It would be of particular value if future research could additionally examine the information on symptom severity and treatment and take their influence into account.In this study we did not conduct a full clinical re-assessment of the patients. Instead, we applied the psychometrically sound broad-band psychopathological questionnaire from ASEBA instruments (ABCL), which was comparable with the childhood information assessed using the CBCL and methodologically sufficient to answer our research question concerning the dimensional modeling of CTD+ADHD. Moreover, we adopted the ABCL fulfilled by the parents instead of self-report from patients to avoid bias from different informants. However, these parent reports need to be interpreted with cautious since the parents are normally not able to observe their children in the same intensity as in the childhood. Four different groups of CTD and/or ADHD patients were investigated according to their assessment at the time of the original study, despite the fact that as young adults some of our patients’ categorization may have changed during development from childhood to young adulthood (e.g. newly onset of tics in former ADHD-only patients). In addition, possible confounding factors such as treatment, severity ratings, and socioeconomic status were not considered. Therefore, interpretations concerning clinical groupings should be treated with caution.

### Conclusion

This 14-year follow-up study tested whether the additive model of childhood CTD+ADHD can also be found in young adulthood. Parent reports supported an interactive model of CTD+ADHD which suggest they perceived the comorbidity CTD+ADHD as a single disorder. In addition, ADHD was no longer the clearly leading problem for psychosocial impairment in comorbid CTD+ADHD in young adulthood. Therefore, the recommendation to treat ADHD first can no longer be given to young adults. Treatment priority should be decided by clinicians on a case-by-case basis depending on the most impairing disorder of each patient, with severity of disorders being considered in further therapeutic decisions.
